# A collection of Constraint Programming models for the three-dimensional stable matching problem with cyclic preferences

**DOI:** 10.1007/s10601-022-09335-y

**Published:** 2022-06-01

**Authors:** Ágnes Cseh, Guillaume Escamocher, Begüm Genç, Luis Quesada

**Affiliations:** 1 Institute of Economics, Centre for Economic and Regional Studies, Budapest, Hungary; 2 Insight Centre for Data Analytics, University College Dublin, Cork, Dublin 4 Ireland; 3 School of Computer Science and Information Technology, University College Cork, Cork, Ireland; 4 Confirm Centre for Smart Manufacturing, Cork, V94 C928 Ireland

**Keywords:** Three-dimensional stable matching with cyclic preferences, 3DSM-cyc, Constraint programming, Fairness

## Abstract

We introduce five constraint models for the 3-dimensional stable matching problem with cyclic preferences and study their relative performances under diverse configurations. While several constraint models have been proposed for variants of the two-dimensional stable matching problem, we are the first to present constraint models for a higher number of dimensions. We show for all five models how to capture two different stability notions, namely weak and strong stability. Additionally, we translate some well-known fairness notions (i.e. sex-equal, minimum regret, egalitarian) into 3-dimensional matchings, and present how to capture them in each model. Our tests cover dozens of problem sizes and four different instance generation methods. We explore two levels of commitment in our models: one where we have an individual variable for each agent (individual commitment), and another one where the determination of a variable involves pairing the three agents at once (group commitment). Our experiments show that the suitability of the commitment depends on the type of stability we are dealing with, and that the choice of the search heuristic can help improve performance. Our experiments not only brought light to the role that learning and restarts can play in solving this kind of problems, but also allowed us to discover that in some cases combining strong and weak stability leads to reduced runtimes for the latter.

## Introduction

In the classic stable marriage problem, we are given a bipartite graph, where the two sets of vertices represent men and women, respectively. Each vertex has a strictly ordered preference list over his or her possible partners. A matching is *stable* if it is not *blocked* by any edge, that is, no man-woman pair exists who are mutually inclined to abandon their partners and marry each other. Stable matchings were first formally defined in the seminal paper of Gale and Shapley [21], who introduced the terminology based on marriage that since then became wide-spread. The notion was then extended to non-bipartite graphs by Irving [28]. Variants of stable matching problems are widely used in employer allocation markets [45], university admission decisions [3, 7], campus housing assignments [8, 43] and bandwidth allocation [20]. Typically, the aim is to solve the decision problem on whether a stable matching exists, or even to solve an optimisation problem considering different fairness notions among stable matchings, such as egalitarian, minimum-regret, or sex-equal.

A natural generalisation of the problem, as suggested by Knuth in his influential book [32], is to extend the two-sided stable marriage problem to three sets of agents. Two input variants of this extension have been defined in the literature. In the first variant, called the *3-gender stable marriage* (3gsm) problem [2, 39], each agent has a preference list over the *n*^2^ pairs of agents from the other two sets, assuming that each agent set contains *n* agents. Another way of generalising stable matching to three agent sets is the *3-dimensional stable matching problem with cyclic preferences* (3dsm-cyc) [39], in which agents from the first set only have preferences over agents from the second set, agents from the second set only have preferences over agents from the third set, and agents from the third set only have preferences over agents from the first set. In both problem variants, the aim is to find a matching that does not admit a blocking triple, where a blocking triple can have slightly different definitions depending on whether the preference lists contain ties or whether a strict improvement for all agents is required. We explore these different notions in Section 1.1.

### 3-dimensional stable matching

In the 3gsm problem variant, the default stability notion is called weak stability, according to which a blocking triple is defined as a set of three agents, all of whom would strictly improve their current match if they would form a triple in the solution. Deciding whether a stable matching exists in a given instance is NP-complete even if the preference lists are complete [39, 49]. A highly restricted preference structure was later identified that allows for a polynomial-time algorithm for the same decision problem [13]. Research then evolved in the direction of preference lists with ties, which gives rise to four different stability definitions, namely weak, strong, super, and ultra stability, and in the direction of consistent preferences, which is a naturally restricted preference domain [27].

The derived research results appear to be more diverse when it comes to the 3dsm-cyc problem variant. Firstly, two stability notions have been investigated: weak and strong. A *weakly stable matching* does not admit a blocking triple such that all three agents would improve, while according to *strong stability*, a triple already blocks if at least one of its agents improves, and the others in the triple remain equally satisfied. Biró and McDermid [5] showed that deciding whether a weakly stable matching exists is NP-complete if preference lists are allowed to be incomplete, and that the same complexity result holds for strong stability even with complete lists. However, the combination of complete lists and weak stability proved to be extremely challenging to solve.

For this setting, Boros et al. [6] proved that each 3dsm-cyc instance admits a weakly stable matching for *n* ≤ 3, where *n* is the size of each vertex set in the tripartition. Eriksson et al. [16] later extended this result to *n* ≤ 4. Additionally, Pashkovich and Poirrier [42] further proved that not only one, but at least two stable matchings exist for each instance with *n* = 5. By this time, the conjecture on the guaranteed existence of a weakly stable matching in 3dsm-cyc with complete lists became one of the most riveting open questions in the matching under preferences literature [32, 34, 52]. Surprisingly, Lam and Plaxton [33] recently disproved this conjecture by showing that weakly stable matchings for 3dsm-cyc need not exist for an arbitrary *n*, moreover, it is NP-complete to determine whether a given 3dsm-cyc instance with complete lists admits a weakly stable matching.

Application-oriented research has focused on the so-called “3-sided matching with cyclic and size preferences” problem, defined by Cui and Jia [12]. They modeled three-sided networking services, such as frameworks connecting users, data sources, and servers. In their setting, users have identical preferences over data sources, data sources have preferences over servers based on the transferred data, and servers have preferences over users. The characterising feature of this variant is that a triple might contain more than one user, as servers aim at maximizing the number of users assigned to them. This feature clearly differentiates the problem from the classic 3dsm-cyc setting. Building upon this work, Panchal and Sharma [41] provided a distributed algorithm that finds a stable solution. Raveendran et al. [44] tested resource allocation in Network Function Virtualisation. They demonstrated the superior performance of the proposed cyclic stable matching framework in terms of data rates and user satisfaction, compared to a centralised random allocation approach.

### Constraint Programming approaches for finding stable matchings

Gent et al. [23] were the first to propose Constraint Programming (CP) models for the classic stable marriage problem. They showed that it is possible to obtain man-optimal and woman-optimal stable matchings immediately from the solution by enforcing Arc Consistency (AC). Later, Unsworth and Prosser [50, 51] presented a binary constraint for the the same problem and showed that their encoding is better in terms of space and time when compared to Gent et al.’s approach. They also investigated sex-equal stable matchings in their studies.

The next milestone was reached by Manlove et al. [35], who proposed three CP models for the Hospital / Residents problem (HR), which is the many-to-one generalisation of the stable marriage problem. They also explored side constraints for their models such as the case with forbidden pairs, residents who may form groups, or residents who may swap their hospitals. The existing research shows that CP models for the stable marriage problem with incomplete lists and for HR are tractable [23, 35]. O’Malley further explored CP models in his thesis for the stable marriage problem, and presented four constraint models [40]. Later on, Siala and O’Sullivan [46] improved the cloned model of Manlove et al. [35] by using a global constraint that achieves Bound Consistency in linear time.

In 2012 Eirinakis et al. [15] used the poset graph of rotations to enumerate all solutions of HR, and presented an improved version of the direct CP model of Manlove et al. [35]. Subsequently, Siala and O’Sullivan [47] used the rotation poset to model stable matchings as SAT formulation for all three types of problems: one-to-one, one-to-many, and many-to-many. They presented empirical results for finding sex-equal stable matchings, and showed that their approach outperforms the model presented in their previous paper [46]. Additionally, Drummond et al. [14] used SAT encoding for finding stable matchings that include couples.

To the best of our knowledge, CP or SAT models for 3-dimensional stable matchings have not been studied before.

## Preliminaries

In this section we introduce the terminology and notation for the problem variants we will study. First we formalise the 3dsm-cyc problem and define the two known stability concepts for it. Then, we define three standard fairness notions that were constructed to distinguish balanced stable solutions on bipartite and non-bipartite stable matching instances.

### 3-dimensional stable matching with cyclic preferences

#### Input and output

Formally, a 3dsm-cyc instance is defined over three disjoint sets of agents of size *n*, denoted by $A= \{ a_{1}, \dots , a_{n} \}$, $B= \{ b_{1}, \dots , b_{n} \}$, and $C= \{ c_{1}, \dots , c_{n} \}$. A *matching* *M* corresponds to a disjoint set of triples, where each triple, denoted by (*a*_*i*_,*b*_*j*_,*c*_*k*_), contains exactly one agent from each agent set. Each agent is equipped with her own preferences in the input. The cyclic property of the preferences means the following: each agent in *A* has a strict and complete preference list over the agents in *B*, each agent in *B* has a strict and complete preference list over the agents in *C*, and finally, each agent in *C* has a strict and complete preference list over the agents in *A*. These preferences are captured by the *rank function*, where $\text {rank}_{a_{i}}(b_{j})$ is the position of agent *b*_*j*_ in the preference list of *a*_*i*_, from 1 if *b*_*j*_ is *a*_*i*_’s most preferred agent to *n* if *b*_*j*_ is *a*_*i*_’s least preferred agent.

#### Preferences over triples

The preference relation of an agent on possible triples can be derived naturally from the preference list of this agent. Agent *a*_*i*_ is indifferent between triples $(a_{i}, b_{j}, c_{k_{1}})$ and $(a_{i}, b_{j}, c_{k_{2}})$, since she only has preferences over the agents in *B* and the same agent *b*_*j*_ appears in both triples. However, when comparing triples $(a_{i}, b_{j_{1}}, c_{k_{1}})$ and $(a_{i}, b_{j_{2}}, c_{k_{2}})$, where $b_{j_{1}}\neq b_{j_{2}}$, *a*_*i*_ prefers the first triple if $\text {rank}_{a_{i}}(b_{j_{1}}) < \text {rank}_{a_{i}}(b_{j_{2}})$, and she prefers the second triple otherwise. The preference relation is defined analogously for agents in *B* and *C* as well.

#### Weak and strong stability

A triple *t* = (*a*_*i*_,*b*_*j*_,*c*_*k*_) is said to be a *strongly blocking triple* to matching *M* if each of *a*_*i*_,*b*_*j*_, and *c*_*k*_ prefer *t* to their respective triples in *M*. Practically, this means that *a*_*i*_,*b*_*j*_, and *c*_*k*_ could abandon their triples to form triple *t* on their own, and each of them would be strictly better off in *t* than in *M*. If a matching *M* does not admit any strongly blocking triple, then *M* is called a *weakly stable* matching. Similarly, a triple *t* = (*a*_*i*_,*b*_*j*_,*c*_*k*_) is called a *weakly blocking triple* if at least two agents in the triple prefer *t* to their triple in *M*, while the third agent does not prefer her triple in *M* to *t*. This means that at least two agents in the triple can improve their situation by switching to *t*, while the third agent does not mind the change. A matching that does not admit any weakly blocking triple is referred to as *strongly stable*. By definition, strongly stable matchings are also weakly stable, but not the other way round. Observe that it is impossible to construct a triple *t* that keeps exactly two agents of a triple equally satisfied, while making the third agent happier, since the earlier two agents need to keep their partners to reach this, which then already defines the triple as one already in *M*.

### Fair stable solutions

In this paper, we translate some standard fairness notions from the classic stable marriage problem to 3dsm-cyc. In most stable matching problems, several stable solutions might be present, which gives way to choosing a fair or balanced one among them. We now review the most prevalent fairness notions in such decisions [10, 26, 30, 34].

#### Egalitarian stable matchings

Possibly the most natural way to define a good stable matching is captured by the notion of *egalitarian stable matchings*. Each agent’s satisfaction can be measured by how high she ranks her partner in the matching. In order to gain a comprehensive measure, we sum up those ranks for all matched agents. A stable matching is called egalitarian, if it minimises this sum among all stable matchings. Finding an egalitarian stable matching in the classic stable marriage problem can be done in polynomial time [25, 29], while it is NP-hard, but 2-approximable if the underlying instance is non-bipartite [18, 19]. A stable matching in a 3dsm-cyc instance that achieves the extreme point of the following function is defined as an egalitarian stable matching.

1 $$\underset{M\text{ is a stable matching}}{\min} \left\{\sum\limits_{(a_{i}, b_{j}, c_{k}) \in M}{\text{rank}_{a_{i}}(b_{j})+ \text{rank}_{b_{j}}(c_{k})+\text{rank}_{c_{k}}(a_{i})}\right\}$$

#### Minimum regret stable matchings

Another popular fairness notion, called *minimum regret stable matching*, intuitively maximises the satisfaction of the least satisfied person in the instance. In this context, each agent’s regret is measured by how high she ranks her partner in the matching—the larger this rank is, the more regret she experiences. The regret of matching *M* is defined as the largest regret in the instance, i.e. the worst rank that appears in the matching. Finding a minimum regret stable matching can be done in polynomial time both in bipartite and non-bipartite instances [24, 25]. A stable matching in a 3dsm-cyc instance that achieves the extreme point of the following function is defined as a minimum regret stable matching.

2 $$\underset{M\text{ is a stable matching}}{\min} \left\{\underset{(a_{i}, b_{j}, c_{k}) \in M}{\max} \left\{\text{rank}_{a_{i}}(b_{j}), \text{rank}_{b_{j}}(c_{k}), \text{rank}_{c_{k}}(a_{i})\right\}\right\}$$

#### Sex-equal stable matchings

A third condition is called *sex-equality*, which aims at reaching the same satisfaction level of each agent set. The satisfaction of a set of agents is measured by summing up the satisfaction level, that is, the rank of the matching partner, of each agent in the set. In the classic stable marriage setting, a sex-equal stable matching minimises the difference between the satisfaction level of the two sets. Finding a sex-equal stable matching is NP-hard in those instances [31, 36]. Even though the notion cannot be defined for non-bipartite instances, it translates readily to 3dsm-cyc instances. The difference of satisfaction level between any two of the three agent sets can be computed exactly as in the classic stable marriage setting. Then, the sum of the three pairwise differences must be minimised. A stable matching in a 3dsm-cyc instance that achieves the extreme point of the following function is defined as a sex-equal stable matching.

3 $$\begin{array}{@{}rcl@{}} &\underset{M\text{ is a stable matching}}{\min} &\left\{ \left\vert\sum\limits_{(a_{i}, b_{j}, c_{k}) \in M}\text{rank}_{a_{i}}(b_{j}) - \text{rank}_{b_{j}}(c_{k})\right\vert \right.\\ &&+\left\vert\sum\limits_{(a_{i}, b_{j}, c_{k}) \in M}\text{rank}_{b_{j}}(c_{k}) - \text{rank}_{c_{k}}(a_{i})\right\vert \\ &&+ \left.\left\vert\sum\limits_{(a_{i}, b_{j}, c_{k}) \in M}\text{rank}_{c_{k}}(a_{i}) - \text{rank}_{a_{i}}(b_{j})\right\vert\right\} \end{array}$$

### Example

**Fig. 1:**
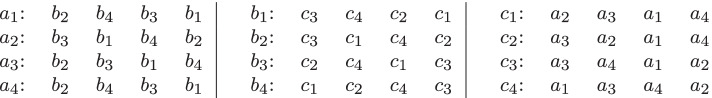
A 3dsm-cyc instance *I* with 4 agents in each agent set

We present in Fig. 1 an example of a 3dsm-cyc instance *I* for *n* = 4. This instance was actually used in our experiments, it comes from the Random dataset. For each agent *a*_*i*_ ∈ *A*, the four agents in *a*_*i*_’s preference list are listed in decreasing order of preference. The same is true for agents from *B* and *C*. For example, *a*_1_’s most preferred, top-choice agent is *b*_2_, her second choice is *b*_4_, her third choice is *b*_3_, and her least preferred, fourth-choice agent is *b*_1_.

**Fig. 2:**
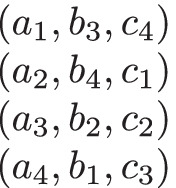
A matching *M* for *I*

We give in Fig. 2 an example of a matching *M* for *I*. Each line in the figure represents a triple of agents who are assigned to each other in *M*. For example, *a*_1_, *b*_3_, and *c*_4_ are assigned to each other. We first explain why *M* is weakly stable. Suppose that there is a strongly blocking triple (*a*_*i*_,*b*_*j*_,*c*_*k*_) for *M*. According to the definition of weak stability, *c*_*k*_ prefers *a*_*i*_ to the agent from *A* she is assigned to in *M*. But *c*_1_, *c*_2_, and *c*_4_ are assigned in *M* to their most preferred agent from *A* (*a*_2_, *a*_3_, and *a*_1_ respectively), while *c*_3_ is assigned to her second-choice agent. So *c*_*k*_ must be *c*_3_, and *a*_*i*_ must be the the agent ranked first by *c*_3_. However, *M* assigns *c*_3_’s first ranked agent *a*_3_ to her top choice *b*_2_. Therefore there can be no strongly blocking triple for *M*, meaning that *M* is weakly stable.

We next show that *M* is not strongly stable. Indeed, consider the triple *t* = (*a*_3_,*b*_2_,*c*_3_). While *a*_3_ is assigned to the same agent from *B* (*b*_2_) in *M* and in *t*, both *b*_2_ and *c*_3_ prefer their assignments in *t* (*c*_3_ and *a*_3_ are both first-choice agents for *b*_2_ and *c*_3_ respectively) over their assignments in *M* (*c*_2_ is last in the preference list of *b*_2_ and *a*_4_ is second in the preference list of *c*_3_). Therefore, from the definition of strong stability, *t* is a weakly blocking triple for *M*, meaning that *M* is not strongly stable.

Let us now look at the cost of *M* according to the fairness notions studied in the paper. Agent *a*_1_ is assigned to her third-choice agent from *B*, *a*_2_ is also assigned to her third-choice agent, *a*_2_ is assigned to her top-choice agent, and *a*_4_ is assigned to her last-choice agent, so the sum of the ranks of the agents from *B* in the preference lists of the agents from *A* whom they are assigned to is 3 + 3 + 1 + 4 = 11. Similarly, the sum of the ranks of the agents from *C* in the preference lists of the agents from *B* whom they are assigned to is 1 + 4 + 2 + 1 = 8, and the sum of the ranks of the agents from *A* in the preference lists of the agents from *C* whom they are assigned to is 1 + 1 + 2 + 1 = 5. The egalitarian cost of *M* is the sum of these three sums, so 11 + 8 + 5 = 24. The minimum regret cost of *M* is 4, because at least one agent are assigned the fourth and last ranked agent in her preference list (*a*_4_ are assigned her least preferred agent *b*_1_). Finally, the sex-equal cost of *M* is the pairwise absolute difference of the sums, so |11 − 8| + |8 − 5| + |5 − 11| = 12.

### Our contribution

This paper is the first to model 3-dimensional stable matchings, specifically the 3dsm-cyc problem, including its optimisation variants using side constraints. We propose the following CP models to find a stable matching in the 3dsm-cyc problem: div (divided agent sets), uni (unified agent sets), and hs (hitting set). We implement each one of the models under both weak and strong stability. For the div and uni models, we investigate two kinds of domain values: one based on the unique identifiers of the agents themselves, referred as *agent-based (agents)*, and the other based on the ranks of agents in one’s preference list, referred as *rank-based (ranks)*. We first use the models to find any satisfying solution to a given 3dsm-cyc instance. Subsequently, we extend all models to optimisation variants under different fairness criteria and conclude with some empirical findings.

## Methodology

In this section we present the details of our five proposed models. For each model, we give the mandatory matching and stability constraints. We also explain how to model the fairness constraints for different optimisation versions. Furthermore, if we identified any, we state the redundant constraints that help the models with better pruning the search space.

### Agent-based DIV model

The div-agents model consists of 3*n* variables $X = \{ x_{1}, \dots , x_{n} \}$, $Y = \{ y_{1},\dots , y_{n} \}$, and $Z = \{ z_{1},\dots ,z_{n} \}$, where the domain of each variable *v* is set as $D(v) = \{1,\dots ,n\}$. For agent-based domain values, assigning *x*_*i*_ = *j* (respectively *y*_*i*_ = *j*, or *z*_*i*_ = *j*) corresponds to matching *a*_*i*_ to *b*_*j*_ (respectively *b*_*i*_ to *c*_*j*_, or *c*_*i*_ to *a*_*j*_). A stable matching *M*, if any exists, is found by using the following constraints.

- (matching) For all 1 ≤ *i*,*j*,*k* ≤ *n*, we add the constraint *x*_*i*_ = *j* ∧ *y*_*j*_ = *k* ⇒ *z*_*k*_ = *i*. This is to ensure that each solution corresponds to a feasible, if not stable, matching.
- (stability) Under *weak* stability, for all 1 ≤ *i*,*j*,*k* ≤ *n*, and for all $i^{\prime },j^{\prime },k^{\prime }$ such that *a*_*i*_ prefers *b*_*j*_ to $b_{j^{\prime }}$, *b*_*j*_ prefers *c*_*k*_ to $c_{k^{\prime }}$ and *c*_*k*_ prefers *a*_*i*_ to $a_{i^{\prime }}$ we add the constraint $x_{i}\neq j^{\prime }\lor y_{j}\neq k^{\prime }\lor z_{k}\neq i^{\prime }$. This is to ensure that there is no strongly blocking triple. When solving the problem under *strong* stability, the condition to post the constraint becomes: *a*_*i*_ prefers *b*_*j*_ to $b_{j^{\prime }}$ or $j^{\prime }=j$, and *b*_*j*_ prefers *c*_*k*_ to $c_{k^{\prime }}$ or $k^{\prime }=k$, and *c*_*k*_ prefers *a*_*i*_ to $a_{i^{\prime }}$ or $i^{\prime }=i$, and $i^{\prime } \neq i \lor j^{\prime } \neq j \lor k^{\prime } \neq k$. Here, as well as in the other models, the difference between weak and strong stability constraints is that the latter also cover the case when exactly two agents of a potential blocking triple are matched together.
- (redundancy) For all 1 ≤ *i*,*j*,*k* ≤ *n*, we add the constraint *y*_*j*_ = *k* ∧ *z*_*k*_ = *i* ⇒ *x*_*i*_ = *j*.
- (redundancy) For all 1 ≤ *i*,*j*,*k* ≤ *n*, we add the constraint *z*_*k*_ = *i* ∧ *x*_*i*_ = *j* ⇒ *y*_*j*_ = *k*.
- (redundancy) We add AllDifferent(*X*) and AllDifferent(*Y* ) and AllDifferent(*Z*) to ensure each agent has exactly one partner from each set.
- (optimisation) When solving a fair version of the problem, we add a constraint to minimise the objective in one of the following ways, depending on which notion of fairness is desired:
  - For egalitarian *M*, we model (1) as: $\sum (\text {rank}_{a_{i}}(b_{j})+\text {rank}_{b_{j}}(c_{k})+\text {rank}{c_{k}}(a_{i}))$ for all *i*,*j*,*k* such that *x*_*i*_ = *j* ∧ *y*_*j*_ = *k* ∧ *z*_*k*_ = *i*.
  - For minimum regret *M*, we model (2) as: $\max \limits (\max \limits (\text {rank}_{a_{i}}(b_{j}),$ $\text {rank}_{b_{j}}(c_{k}),$ rank*c*_*k*_(*a*_*i*_))) for all *i*,*j*,*k* such that *x*_*i*_ = *j* ∧ *y*_*j*_ = *k* ∧ *z*_*k*_ = *i*.
  - For sex-equal *M*, we model (3) as: $\left \vert S_{A}-S_{B}\right \vert +\left \vert S_{B}-S_{C}\right \vert +\left \vert S_{C}-S_{A}\right \vert$ where $S_{A}=\sum (\text {rank}_{a_{i}}(b_{j}))$ for all *i*,*j* such that *x*_*i*_ = *j*, $S_{B}=\sum (\text {rank}_{b_{j}}(c_{k}))$ for all *j*,*k* such that *y*_*j*_ = *k*, and $S_{C}=\sum (\text {rank}_{c_{k}}(a_{i}))$ for all *k*,*i* such that *z*_*k*_ = *i*.

### Rank-based DIV model

Variables and domains are the same in the rank-based div model (div-ranks) as they are in the agent-based div model (div-agents), but this time assigning *x*_*i*_ = *j* (respectively *y*_*i*_ = *j*, or *z*_*i*_ = *j*) corresponds to matching *a*_*i*_ to her *j*^th^ preferred agent (respectively *b*_*i*_ to her *j*^th^ preferred agent, or *c*_*i*_ to her *j*^th^ preferred agent), who might be different from *b*_*j*_. A stable matching *M*, if any exists, is found by using the following constraints.

- (matching) For all 1 ≤ *i*,*j*,*k* ≤ *n*, we add the constraint $x_{i}=\text {rank}_{a_{i}}(b_{j})\land y_{j}=\text {rank}_{b_{j}}(c_{k})\Rightarrow z_{k}=\text {rank}_{c_{k}}(a_{i})$. This is to ensure that each solution corresponds to a feasible, if not stable, matching.
- (stability) Under *weak* stability, for all 1 ≤ *i*,*j*,*k* ≤ *n*, we add the constraint $x_{i}\leq \text {rank}_{a_{i}}(b_{j})\lor y_{j}\leq \text {rank}_{b_{j}}(c_{k})\lor z_{k}\leq \text {rank}_{c_{k}}(a_{i})$. This is to ensure that there is no strongly blocking triple. When solving the problem under *strong* stability, the inequalities are strict but the following part is added to each disjunction: $\lor (x_{i} = \text {rank}_{a_{i}}(b_{j})\land y_{j} = \text {rank}_{b_{j}}(c_{k})\land z_{k} = \text {rank}_{c_{k}}(a_{i}))$.
- (redundancy) For all 1 ≤ *i*,*j*,*k* ≤ *n*, we add the constraint $y_{j}=\text {rank}_{b_{j}}(c_{k})\land z_{k}=\text {rank}_{c_{k}}(a_{i})\Rightarrow x_{k}=\text {rank}_{a_{i}}(b_{j})$.
- (redundancy) For all 1 ≤ *i*,*j*,*k* ≤ *n*, we add the constraint $z_{k}=\text {rank}_{c_{k}}(a_{i})\land x_{i}=\text {rank}_{a_{i}}(b_{j})\Rightarrow y_{j}=\text {rank}_{b_{j}}(c_{k})$.
- (optimisation) We add a constraint to minimise the objective in one of the following ways, depending on which notion of fairness is desired:
  - For egalitarian *M*, we model (1) as: ${\sum }_{i=1}^{n}(x_{i}+y_{i}+z_{i})$.
  - For minimum regret *M*, we model (2) as: $\max \limits (\max \limits (x_{i},y_{i},z_{i}))$ for all 1 ≤ *i* ≤ *n*.
  - For sex-equal *M*, we model (3) as: $\left \vert S_{A}-S_{B}\right \vert +\left \vert S_{B}-S_{C}\right \vert +\left \vert S_{C}-S_{A}\right \vert$ where $S_{A}={\sum }_{i=1}^{n}(x_{i})$, $S_{B}={\sum }_{j=1}^{n}(y_{j})$, and $S_{C}={\sum }_{k=1}^{n}(z_{k})$.

Note that, as opposed to agent-based domains, there are no AllDifferent constraints in rank-based models. The reason for this is that with rank-based domains it is possible for two agents in the same agent set to be assigned the same value, for example if they both got assigned to their most preferred agent.

### Agent-based UNI model

The uni-agents model consists of *n* variables $X=\{x_{1},\dots ,x_{n}\}$, where the domain of each variable *v* is set as $D(v)=\{(1,1),\dots ,(1,n),(2,1),\dots ,(n,n)\}$. Each tuple domain variable is implemented as an integer domain variable by representing the tuple (*j*,*k*) with the integer (*j* − 1)*n* + *k*. For agent-based domain values, assigning (*j*,*k*) to *x*_*i*_ corresponds to having the triple (*a*_*i*_,*b*_*j*_,*c*_*k*_) in the matching. A stable matching *M*, if any exists, is found by using the following constraints.

In both the current and following subsections, we denote by *x*_*i*,*B*_ and *x*_*i*,*C*_ respectively the first and second elements of the pair assigned to *x*_*i*_.

- (matching) For all $1\leq i<i^{\prime }\leq n$, we add the constraint $x_{i,B}\neq x_{i^{\prime },B}\land x_{i,C}\neq x_{i^{\prime },C}$. This is to ensure that each solution corresponds to a feasible, if not stable, matching.
- (stability) Under *weak* stability, for all 1 ≤ *i*,*j*,*k* ≤ *n*, for all $1\leq i^{\prime },i^{\prime \prime },j^{\prime },k^{\prime }\leq n$ such that *a*_*i*_ prefers *b*_*j*_ to $b_{j^{\prime }}$, *b*_*j*_ prefers *c*_*k*_ to $c_{k^{\prime }}$ and *c*_*k*_ prefers *a*_*i*_ to $a_{i^{\prime }}$, we add the constraint $(x_{i,B}\neq j^{\prime })\lor (x_{i^{\prime \prime }}\neq (j,k^{\prime }))\lor (x_{i^{\prime },C}\neq k)$. This is to ensure that no triple is strongly blocking. Because in UNI only the agents from *A* have their own associated variables, determining whether *b*_*j*_ was assigned to $c_{k^{\prime }}$ requires checking for each agent $a_{i^{\prime \prime }}$ from *A* whether both *b*_*j*_ and $c_{k^{\prime }}$ were assigned to $a_{i^{\prime \prime }}$. This is the reason for the additional index $i^{\prime \prime }$. When solving the problem under *strong* stability, the condition to post the constraint becomes: *a*_*i*_ prefers *b*_*j*_ to $b_{j^{\prime }}$ or $j^{\prime }=j$, and *b*_*j*_ prefers *c*_*k*_ to $c_{k^{\prime }}$ or $k^{\prime }=k$, and *c*_*k*_ prefers *a*_*i*_ to $a_{i^{\prime }}$ or $i^{\prime }=i$, and $i^{\prime } \neq i \lor j^{\prime } \neq j \lor k^{\prime } \neq k$.
- (redundancy) We impose the constraint AllDifferent(*X*).
- (redundancy) Denote by *F*(*i*) the set of tuples that have an agent *i* as their first element, and *S*(*i*) the tuples that have *i* as their second. Then, for all agents ∀*i* ∈ *n* we have ${\sum }_{j \in F(i)} \textsc {Count}(j, X) = 1$ and ${\sum }_{j \in S(i)} \textsc {Count}(j, X) = 1$.
- (optimisation) We add a constraint to minimise the objective in one of the following ways, depending on which notion of fairness is desired:
  - For egalitarian *M*, we model (1) as: ${\sum }_{i=1}^{n}(\text {rank}_{a_{i}}(b_{x_{i,B}})+\text {rank}_{b_{x_{i,B}}}(c_{x_{i,C}})+\text {rank}_{c_{x_{i,C}}}(a_{i}))$.
  - For minimum regret *M*, we model (2) as: $\max \limits (\max \limits ($ $\text {rank}_{a_{i}}(b_{x_{i,B}}),$ $\text {rank}_{b_{x_{i,B}}}(c_{x_{i,C}}),\text {rank}_{c_{x_{i,C}}}(a_{i})))$ for all 1 ≤ *i* ≤ *n*.
  - For sex-equal *M*, we model (3) as: $\left \vert S_{A}-S_{B}\right \vert +\left \vert S_{B}-S_{C}\right \vert +\left \vert S_{C}-S_{A}\right \vert$ where $S_{A}={\sum }_{i=1}^{n}(\text {rank}_{a_{i}}(b_{x_{i,B}}))$, $S_{B}={\sum }_{i=1}^{n}(\text {rank}_{b_{x_{i,B}}}(c_{x_{i,C}}))$, and $S_{C}={\sum }_{i=1}^{n}(\text {rank}_{c_{x_{i,C}}}(a_{i}))$.

### Rank-based UNI model

Variables and domains are implemented the same in the rank-based uni model (uni-ranks) as they are in the agent-based uni model (uni-agents), but this time assigning (*j*,*k*) to *x*_*i*_ corresponds to matching *a*_*i*_ to her *j*^th^ preferred agent from *B*, and matching the latter to her *k*^th^ preferred agent from *C*. A stable matching *M*, if any exists, is found by using the following constraints.

- (matching) For all $1\leq i<i^{\prime }\leq n$, we add the constraint $\text {pref}_{a_{i}}(x_{i,B})\neq \text {pref}_{a_{i^{\prime }}}(x_{i^{\prime },B})\land \text {pref}_{\text {pref}_{a_{i}}(x_{i,B})}(x_{i,C})\neq \text {pref}_{\text {pref}_{a_{i^{\prime }}}(x_{i^{\prime },B})}(x_{i^{\prime },C})$, where $\text {pref}_{a_{i}}(r)$ (respectively $\text {pref}_{b_{j}}(r)$, or $\text {pref}_{c_{k}}(r)$) represents the agent *b* ∈ *B* (respectively *c* ∈ *C*, or *a* ∈ *A*) such that $\text {rank}_{a_{i}}(b)=r$ (respectively $\text {rank}_{b_{j}}(c)=r$, or $\text {rank}_{c_{k}}(a)=r$). This is to ensure that each solution corresponds to a feasible, if not stable, matching.
- (stability) Under *weak* stability for all 1 ≤ *i*,*j*,*k* ≤ *n*, for all $1\leq i^{\prime },i^{\prime \prime },j^{\prime \prime }\leq n$ such that *c*_*k*_ strictly prefers *a*_*i*_ to $a_{i^{\prime }}$, we add the constraint $(x_{i,B}\leq \text {rank}_{a_{i}}(b_{j}))\lor (x_{i^{\prime \prime },B}\neq \text {rank}_{a_{i^{\prime \prime }}}(b_{j}))\lor (x_{i^{\prime \prime },C}\leq \text {rank}_{b_{j}}(c_{k}))\lor (x_{i^{\prime }}\neq (\text {rank}_{a_{i^{\prime }}}(b_{j^{\prime \prime }}),\text {rank}_{b_{j^{\prime \prime }}}(c_{k})))$. This is to ensure that no triple is strongly blocking. When solving the problem under *strong* stability, *c*_*k*_’s preference of *a*_*i*_ to $a_{i^{\prime }}$ is not strict ($i^{\prime }$ can be equal to *i*) but the two inequalities are, and to each disjunction is added the following part: $\lor (x_{i}=(\text {rank}_{a_{i}}(b_{j}),\text {rank}_{b_{j}}(c_{k})))$.
- (optimisation) We add a constraint to minimise the objective in one of the following ways, depending on which notion of fairness is desired:
  - For egalitarian *M*, we model (1) as: ${\sum }_{i=1}^{n}(x_{i,B}+x_{i,C}+\text {rank}_{\text {pref}_{\text {pref}_{a_{i}}(x_{i,B})}(x_{i,C})}(a_{i}))$.
  - For minimum regret *M*, we model (2) as: $\max \limits (\max \limits (x_{i,B},x_{i,C},$ $\text {rank}_{\text {pref}_{\text {pref}_{a_{i}}(x_{i,B})}(x_{i,C})}(a_{i})))$ for all 1 ≤ *i* ≤ *n*.
  - For sex-equal *M*, we model (3) as: $\left \vert S_{A}-S_{B}\right \vert +\left \vert S_{B}-S_{C}\right \vert +\left \vert S_{C}-S_{A}\right \vert$ where $S_{A}={\sum }_{i=1}^{n}(x_{i,B})$, $S_{B}={\sum }_{i=1}^{n}(x_{i,C})$, and $S_{C}={\sum }_{i=1}^{n}(\text {rank}_{\text {pref}_{\text {pref}_{a_{i}}(x_{i,B})}(x_{i,C})}(a_{i}))$.

### HS model

In the hs model, let *T* be the set of all possible triples as {(1,1,1),(1,1,2),…,(*n*,*n*,*n*)}. Without loss of generality, assume that the triples in *T* are ordered, so *t*_*i*_ ∈ *T* refers to the *i*^th^ triple of *T*. Given a triple *t* ∈ *T*, we denote by *BT*(*t*) all the triples in *T* that prevent *t* from becoming a blocking triple given the preferences. Then, finding a stable matching is equivalent to finding a hitting set of the non-blocking triples in *T*.

- Let *M* be a set variable whose upper bound is {*i* : *t*_*i*_ ∈ *T*}.
- (matching) Ensure that each agent from each set is matched by having:
  - $$\forall a \in A : {\sum }_{t_{i} \in T : a \in t_{i}} (i \in M) =1$$;
  - $$\forall b \in B : {\sum }_{t_{i} \in T : b \in t_{i}} (i \in M) =1$$;
  - $$\forall c \in C : {\sum }_{t_{i} \in T : c \in t_{i}} (i \in M) =1$$.
- (stability) The stable matching is a hitting set of the non-blocking triples: ∀*t*_*j*_ ∈ *T* : *M* ∩{*i* : *t*_*i*_ ∈*BT*(*t*_*j*_)}≠*∅*. The type of stability is addressed in the computation of the *BT* sets. The model as such is not concerned with this aspect.

In this model, *M* is constrained to be a set of triples representing the stable matching as defined in Section 2.2, so egalitarian *M*, minimum regret *M*, and sex-equal *M* are defined as in (1), (2), and (3) respectively.

In the actual implementation, *M* is represented in terms of an array of *n*^3^ Boolean variables, where each variable refers to the inclusion/exclusion of the corresponding tuple in the mapping.

## Experiments

We performed our experiments on machines with Intel(R) Xeon(R) CPU with 2.40GHz running on Ubuntu 18.04. Our initial experiments on small instances comparing all models are performed using Gecode 6.3.0 [22]. Then, we conducted further experiments by using our best performing models for larger instances on a constraint solver based on lazy-clause generation, namely Chuffed 0.10.4. [9]. For div and uni models, instances were first processed by MiniZinc 2.5.5 [38] before being given to the solvers. The hs model was directly encoded using Gecode 6.2.0.

In Section 4.1 we describe the datasets in use. In Section 4.2, we explain how we chose the heuristic used in our experiments, and compare activity-based heuristics against non-activity based ones. In Section 4.3 we compare the proposed models, before testing the best performing one on large instances in Section 4.4. We then explore an alternative way to model weak stability in Section 4.5.

### Dataset description

For every size *n* present in our experiments, we generated 100 instances with *n* agents in each agent set and a complete list for each agent. Half of these instances are random and the other half have some or all of the preferences based on master lists. Master list instances are instances where the preference lists of all agents in the same agent set are identical. Master lists provide a natural way to represent the fact that in practice agent preferences are often not independent. Examples of real-life applications of master lists occur in resident matching programs [4], dormitory room assignments [43], cooperative download applications such as BitTorrent [1], and 3-sided networking services [12]. The detailed distribution of the 100 instances generated for each size is as follows:

- **Random**: 50 random instances from a uniform distribution.
- **ML_oneset**: 20 instances where the preference lists of the agents in one of the agent sets are based on master lists, and the preference lists of the agents in the other two agent sets are random.
- **ML_1swap**: 15 instances, where each agent set has a randomly chosen master list that all agents in the set follow. Then, we randomly choose two agents from each agent’s preference list, and swap their positions.
- **ML_2swaps**: 15 instances, where each agent set has a randomly chosen master list that all agents in the set follow. First, we randomly choose two agents from each agent’s preference list, and swap their positions. Subsequently, we randomly choose two more agents from each list such that the new agents were not involved in the first swap, then we swap their positions.

Note that neither the type of stability (weak or strong) nor the fairness objective is part of the preferences themselves. For this reason, the 100 instances generated for each size *n* are used for both types of stability and for all satisfiability and optimisation versions.

We did not consider instances where all preference lists in all three sets are exact master lists, because the complete set of stable matchings for these instances is known [17]. We show in Lemma 1 that ML_oneset instances always have a strongly stable matching, but, contrary to the case with master lists in all three sets, not all solutions have been characterised. Therefore there is value in modelling fairness versions of the problem for instances with this structure.

#### Lemma 1

Each 3dsm-cyc instance with complete lists and one master list admits at least one strongly stable matching.

#### Proof

Assume that agent set *C* is equipped with a master list. For each agent *c*_*k*_ ∈ *C*, let us relabel the preferred agents of *c*_*k*_ from set *A* in her preference list so that $\text {rank}_{c_{k}}(a_{i}) < \text {rank}_{c_{k}}(a_{i+1})$ for each 1 ≤ *i* ≤ *n* − 1. We run a serial dictatorship algorithm as follows. First *a*_1_ chooses her first choice agent in *B*, who then chooses her first choice agent in *C*. This triple is removed from the instance. Then we iterate the same starting with *a*_2_, who chooses her first choice among agents in *B* not yet removed, and so on. Let us relabel agents in *B* and *C* so that triples (*a*_*i*_,*b*_*i*_,*c*_*i*_) form the output matching of this algorithm.

First, observe that an agent *a*_*i*_ can only prefer an agent *b*_*j*_ to her partner in *M* if *j* < *i*. Similarly, *a*_*i*_ prefers *b*_*i*_ to all agents with *j* > *i*. These observations hold for agents *b*_*i*_ and *c*_*i*_ as well. Each weakly blocking triple thus must include a decrease in the variable index where the strict preference occurs, and simultaneously can contain no decrease in the index elsewhere, because this would make the corresponding agent less satisfied as she was in *M*. 

### Heuristic selection

We compared the uni and div models using various built-in search strategies on constraint solvers: Gecode and Chuffed. There are two key factors that may have significant impact on the performance of a search algorithm for constraint satisfaction. These are namely *variable ordering* and *value ordering* heuristics. The variable ordering heuristics define an order in which variables are chosen for instantiation during the search. Similarly, the value ordering heuristics define the decision-making procedure behind selecting which value to assign to an already selected variable. The ordering may have significant effect on the complexity of the backtrack search and the size of the search tree in terms of its branches.

In our experiments, we used three built-in variable ordering heuristics (also known as variable selection annotation in Minizinc documentation) from Minizinc listed as: *input_order (none), first_fail (fail), smallest (smallest)*. These heuristics are defined as: searching variables in the given order, choosing the variable with the smallest domain, and choosing the variable with the smallest value in its domain, respectively. The given order is defined as the ordering of variables we provide to the solver, i.e. from *x*_1_ to *x*_*n*_, then from *y*_1_ to *y*_*n*_, then from *z*_1_ to *z*_*n*_ for div-agents and div-ranks; from *x*_1_ to *x*_*n*_ for uni-agents and uni-ranks; and from *t*_1_ to $t_{n^{3}}$ for hs. Note that we provided, in parentheses, the abbreviations that we used throughout this paper to refer to these strategies. We also used four built-in value ordering heuristics (also known as value choice annotations) listed as: *indomain_min (min), indomain_median (median), indomain_max (max), and indomain_split (split)* [48]. These value ordering heuristics in order are defined as: assigning the smallest, middle, or largest value in the domain, and bisecting the domain by excluding the upper half first, respectively. The ordering heuristics above were chosen as they are available for both Gecode and Chuffed. Additionally, in Gecode, we tested our models using two more variable ordering heuristics: *action_size_max (size)* that selects a variable with the largest activity divided by its domain size, and *action_max (actmax)* that selects the variable with the highest activity. We also explored the effect of *free search (free)* option offered by the Chuffed solver, which allows this solver to ignore any search annotations and to revert to an activity-based approach. Table 1 lists all the heuristic combinations that we tested for each problem type.

**Table 1:** The set of search heuristics used in the experiments for each configuration

Solver	Model	Search heuristic
Gecode	{div-, uni-}x{agents, ranks}	{fail, none, size, smallest}x{min, median, max, split}
Gecode	hs-eager	{actmax, none} x {max, min}
Chuffed	{div-, uni-}x{agents, ranks}	{freefail, fail, none, smallest}x{min, median, max, split}

Each row covers both stability notions: weak and strong

In the rest of this section, we refer to each variable, value ordering heuristic combination as the *search heuristic*. The search heuristic is labelled with the combined abbreviations of the relevant annotations, i.e. the abbreviation of the variable ordering heuristic followed by the abbreviation of the value ordering heuristic. For the sake of demonstration, smallestmax strategy corresponds to a combination of the variable ordering heuristic ‘smallest’ and the value ordering heuristic ‘indomain_max’. Similarly, failsplit corresponds to using a combination of ‘first_fail’ and ‘indomain_split’ heuristics. In total, we carried out an extensive testing stage for our models by using 268 different configurations, i.e. different values for solver, model, method, stability, search heuristic.

It is important to note here that we implemented the div and uni models in Minizinc, which allows us to solve the problem using both Gecode and Chuffed solvers. Subsequently, we implemented these models natively in Gecode and Chuffed to observe if there are any advantages or disadvantages. For our third model, hs, we implemented it natively in Gecode and also natively in Chuffed. In this case, we avoided the implementation of hs in Minizinc due to large number of tuples to be pre-processed. We observed that our native implementations for div and uni model perform worse when compared to the Minizinc implementations. It is our belief that Minizinc may have some solver-specific settings that we have not applied in our native implementations. In conclusion, we used Minizinc implementations of div and uni models, and native Gecode implementation of the hs model to perform the experiments detailed in the rest of this section.

For all our the experiments, we used a time limit of 10 minutes for each instance. Considering the huge number of all parameter configurations for each model, we adapted a look-ahead approach for our tests. To elaborate further on this, we performed our tests for each one of our models starting with small instances (i.e. *n* = 4), then we incremented the size for each model that was promising up to *n* = 16. We considered a model as promising, if it did not time out for the majority of the instances for the given size, and its performance is better (or comparable) to another model for the same size. Out of all tested values of *n*, we selected four values that allow us to clearly observe the behaviour, and also the top-four best-performing search heuristics for each configuration for readability.

In our previous conference paper [11], we did not explore solver-specific search heuristics ‘size’, ‘actmax’, and ‘free’ for our models. Table 2 presents the previously reported best (not activity-based) heuristic for each model, solver, and stability. We extended our experiments to include activity-based heuristics to compare their performance with the previously reported best-performing heuristics.

**Table 2:** A study of whether using an activity-based heuristic leads to a better performance, depending on the model, solver, and stability

Model	Solver	Stability	Best (not activity-based) heuristic	Best heuristic
div-agents	Gecode	weak	failmin	**sizemaxmedian**
div-agents	Gecode	strong	failmin	**sizemaxmin**
div-agents	Chuffed	weak	failmin	**freefailmax**
div-agents	Chuffed	strong	failmin	**freefailmax**
div-ranks	Gecode	weak	failmin	**sizemaxmin**
div-ranks	Gecode	strong	**failmin**	failmin
div-ranks	Chuffed	weak	failmin	**freefailsplit**
div-ranks	Chuffed	strong	failmin	**freefailsplit**
uni-agents	Gecode	weak	**failsplit**	failsplit
uni-agents	Gecode	strong	**failmin**	failmin
uni-agents	Chuffed	weak	failsplit	**freefailmin**
uni-agents	Chuffed	strong	**failsplit**	failsplit
uni-ranks	Gecode	weak	**failsplit**	failsplit
uni-ranks	Gecode	strong	**failmin**	failmin
uni-ranks	Chuffed	weak	**failsplit**	failsplit
uni-ranks	Chuffed	strong	**failsplit**	failsplit
hs	Gecode	weak	nonemax	**actmaxmax**
hs	Gecode	strong	nonemax	**actmaxmax**

Bold entries indicate whether the best performance was obtained with an activity-based heuristic


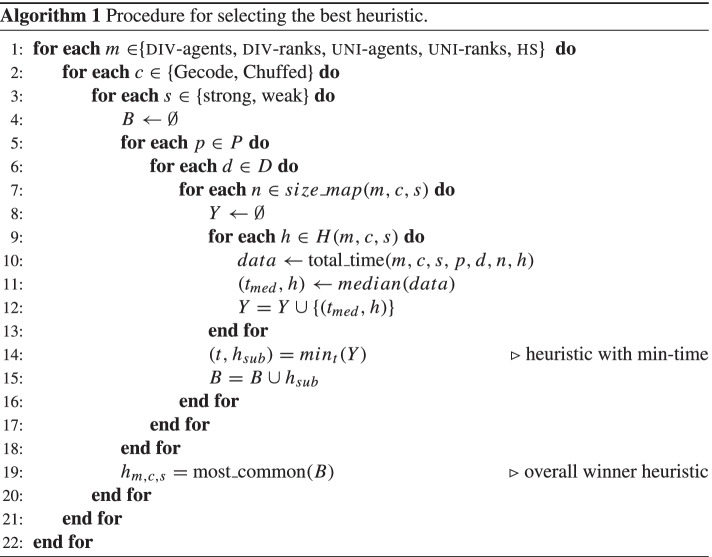


In order to find the best performing heuristic for each model in the extended set, we analysed the performances of each search heuristic by the total time spent from running the model until obtaining a result, and also the number of failures encountered during search for each instance. We used Algorithm 1 to analyse all our configurations to select the best-performing heuristic to be used for the rest of our experiments. The algorithm analyses for each (model, solver, stability) the performance of each heuristic on each problem type (e.g. satisfiability, minimum regret, sex-equal, egalitarian) and each family of instances (e.g. Random, ML_oneset, ML_1swap, ML_2swaps). In this evaluation, we used different subsets of values of *n*: {{5,7,9,11},{4,8,12,16},{5,7,8,12}} such that the values let us clearly observe the difference. We define the best-performing search heuristic as the most common winner of the sub-tests for each (model, solver, stability). In this algorithm, *P* denotes the set of the problems, where *P* = {satisfiability, egalitarian, sex-equal, minimum regret}, and *D* denotes the set of all data generation methods, where *D* = {Random, ML_oneset, ML_1swap, ML_2swaps}. We denote by *B* the set of identifiers of all sub-winner search heuristics for each (model, solver, stability). We define by *Y* a set of tuples, where each tuple (*t*,*h*) corresponds to the median of the total time *t* spent by a heuristic identified by *h* to solve all instances for any configuration: (model, solver, stability, problem, dataset, size). For each such configuration, the sub-winner search heuristic *h*_*sub*_ is identified as the one that has the minimum median value of the total time *t* among all tuples in *Y*, using Line 8 to Line 15 in the procedure. In Line 17, we use *size*_*map*(*m*,*c*,*s*) as the set of four values of *n* selected for each model *m*, solver *c*, and stability *s*. Similarly, in Line 9, *H*(*m*,*c*,*s*) denotes the set of unique identifiers of the top four heuristics for any given model *m*, solver *c*, and stability *s*.

We present the best-performing search heuristic for each configuration in the fifth column of Table 2. While activity-based heuristics did not help the performance of either uni model, they almost always led to improvements for both div models, as well as for hs. In the next subsection we will show that hs is the best performing model for weak stability using Gecode, and that for the other three (solver, stability) combinations it is div-ranks. This means that activity-based heuristics allowed us to see reduced runtimes from the winning model in three out of the four (solver, stability) configurations that we studied: hs in (Gecode, weak), and div-ranks in (Chuffed, strong) and (Chuffed, weak).

**Fig. 3:**
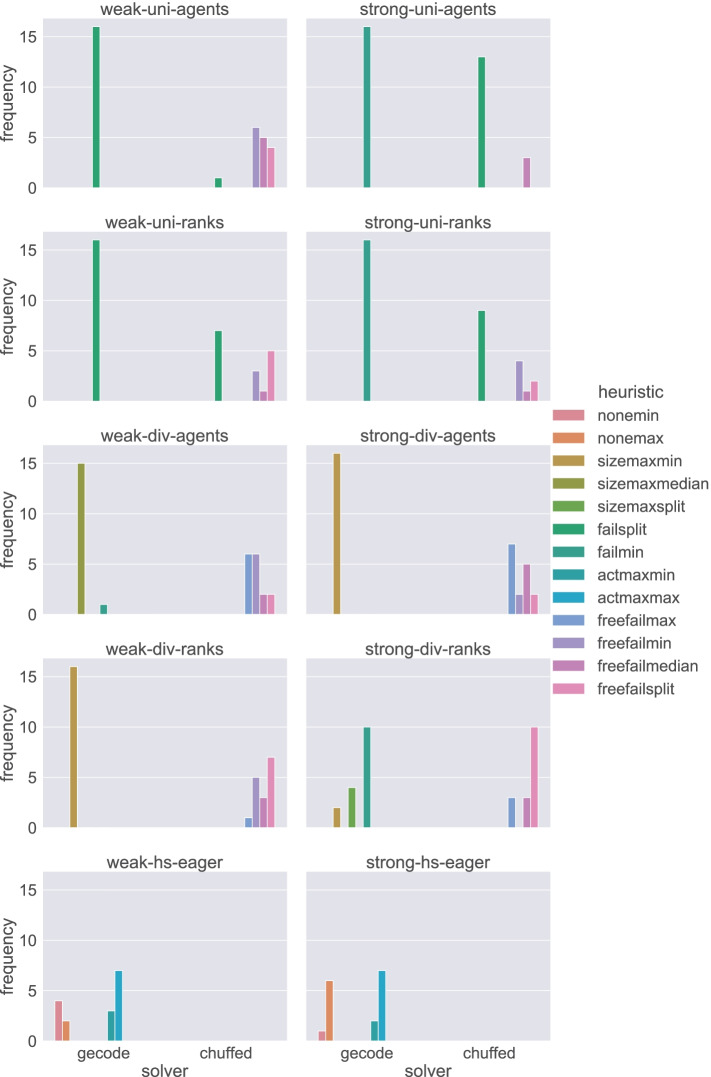
Comparing the performance of the heuristics in *H*(*m*,*c*,*s*) for every *m* ∈{div-agents, div-ranks, uni-agents, uni-ranks, hs}, *c* ∈{Gecode, Chuffed} and *s* ∈{strong, weak}

In Fig. 3 we compare the performance of the heuristics in *H*(*m*,*c*,*s*) for every *m* ∈{div-agents, div-ranks, uni-agents, uni-ranks, hs}, *c* ∈{Gecode, Chuffed} and *s* ∈{strong, weak}. For every *h* in *H*(*m*,*c*,*s*) we plot the number of times *h* appears as the winner. That is, the heuristic with the highest frequency is the most common heuristic for (*m*,*c*,*s*) (i.e. *most*_*common*(*B*)). In the Gecode experiments, the winner was very clear in most cases. There was only one case (hs under strong stability) where the race for the first place was close. In quite a few cases there was only one option in Gecode. In Chuffed we observed more competition between the alternatives, which suggests that in Chuffed the dependency on the heuristics seems less significant.

### Model comparison by time

In this section, subsequent to testing all configurations in Section 4.2 as presented in Table 1 and using the best heuristics found, we compare the relative performances of our five models (i.e. hs, div-agents, div-ranks, uni-agents, and uni-ranks) on small instances in Gecode and Chuffed. Our intention for this comparison is to find out which strategy (e.g. model, method, solver) performs best. Note that in Gecode experiments, we did not use extra propagation techniques such as lazy clause generation or restarts.

To provide a comprehensive analysis of our tests, we used notched boxplots [37] for most of the plots in this section. In notched boxplots, the narrowing of the box indicates the median of the data, and the notches surrounding the medians provide a measure of significance of difference. Thus, not overlapping notches around the medians suggest a notable difference.

**Fig. 4:**
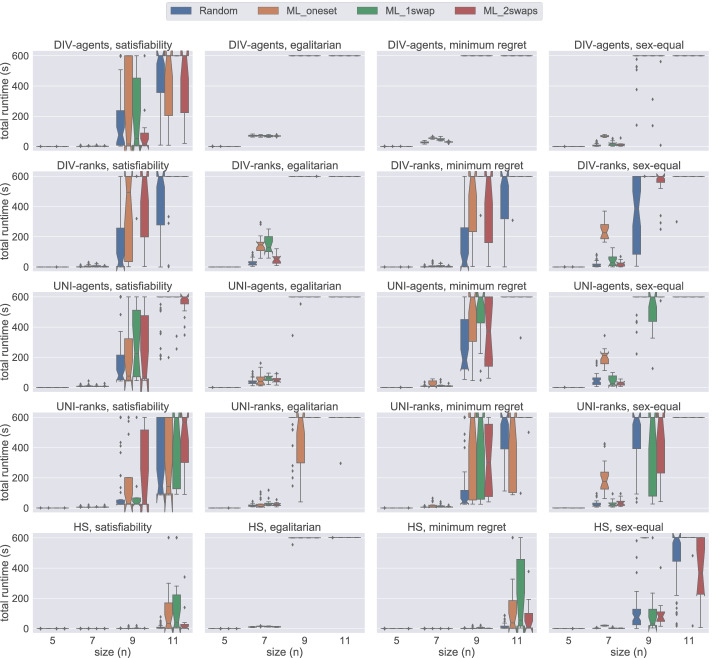
A comparison of total time spent by all models under weak stability using Gecode

Figure 4 presents a comparison of total time required by all five models on small instances of size 5 ≤ *n* ≤ 11 under weak stability solved using Gecode. The first insight that can be gained from this figure is that the hs model handles the instances of small sizes very well. When we examine performance based on the dataset generation methods, we observe that usually a weakly stable matching for the instances in Random is found faster than for other datasets when using Gecode. Additionally we observe that the proposed models require more time to solve the instances in ML_1swap for satisfiability, egalitarian, and minimum regret versions. On the other hand, for the sex-equal version of the problem, ML_oneset is the most challenging dataset for all models. From this figure, we conclude that hs is the best model when dealing with small instances under weak stability using Gecode.

**Fig. 5:**
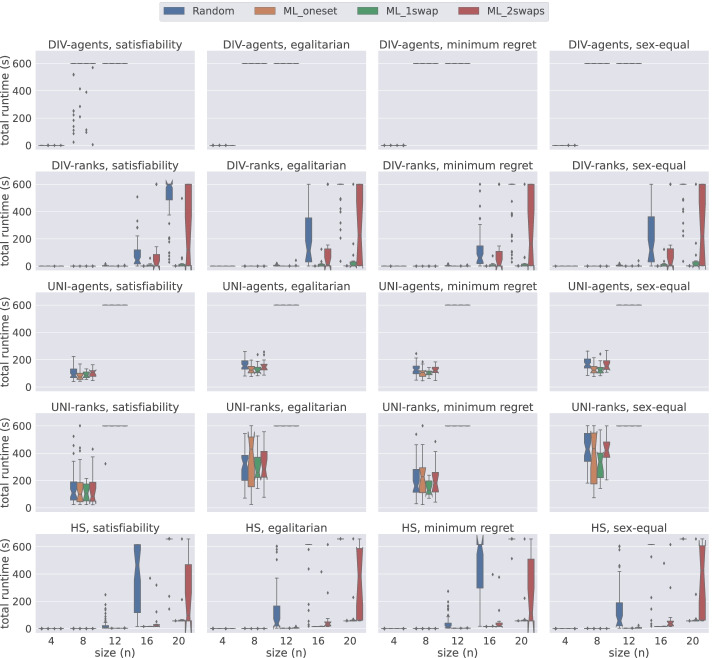
A comparison of total time spent by all models under strong stability using Gecode

Figure 5 demonstrates the results obtained from the same datasets under strong stability. Note that the problem model for finding a matching under weak stability is a relaxed version of the model under strong stability. However, on average, strongly stable matchings are found faster than their weak counterpart. We can clearly observe this behaviour in Fig. 5. All models except div-agents were able to solve all satisfiable instances of size between 4 ≤ *n* ≤ 11 within the given time limit. Therefore, in order to provide more insight into the performance of models, we use a larger scale, i.e. {4,8,12,16,20} in Fig. 5. Under strong stability, we clearly observe that hs and div-ranks scale better when compared to the other models. For instance, for the satisfiability problem with size *n* = 8, both hs and div-ranks quickly solve all the instances. However, both uni-agents and uni-ranks require longer time than hs and div-ranks, whereas div-agents fails to solve many instances within the given time limit. Both uni and hs follow the same commitment approach (group commitment). We believe this is hampering their scalability as there are fewer solutions in the strong stability case, which increases the chances of making wrong choices thus leading to higher penalties in the case of group commitment as we have to undo the three pairings. Considering that the time performance of the uni models and div-agents are considerably worse for 4 ≤ *n* ≤ 12 when compared to others, we decided to discard them from further experiments. hs is not performing that bad, but its scalability is affected by the computation of the BT sets. The size of each BT set is *O*(*n*^3^), which is a remarkable overhead when dealing with big instances. We elaborate more on this limitation in Section 4.4. Therefore, we conclude from this figure that div-ranks is the most efficient model when working with large *n* under strong stability using Gecode.

Note that the hs model was implemented in Gecode directly because of the cost of its preprocessing phase. It is cheaper to carry out the computation of the BT sets in C++ than in MiniZinc. However, this decision does not put the other models in a disadvantageous position since the main contribution to the running time comes from the solving time and in all cases the solving phase is carried out in C++. The assumption here is that we are dealing with small sizes where the flattening phase is not considered a significant burden.

In addition to the Gecode experiments, we tested our four models div-agents, div-ranks, uni-agents, and uni-ranks on Chuffed. Chuffed is the state-of-the art lazy clause solver that performs propagation by recording the reasons of propagation at each step. This helps with efficiently creating nogoods during the search and avoiding failures. Note that due to implementing the hs model directly in Gecode, our Chuffed experiment results do not include the performance of the hs model. It is certainly possible to implement the hs model in Chuffed directly. In fact, we did so but the performance observed was considerably worse than the one we obtained with Gecode. We acknowledge that we did not achieve a deep understanding of the C++ application programming interface of Chuffed, so we cannot claim that we used Chuffed in the most efficient way when encoding our models directly in Chuffed. However, there is a chance that Chuffed might be used in a more efficient way with a deeper understanding of its C++ application programming interface.

**Fig. 6:**
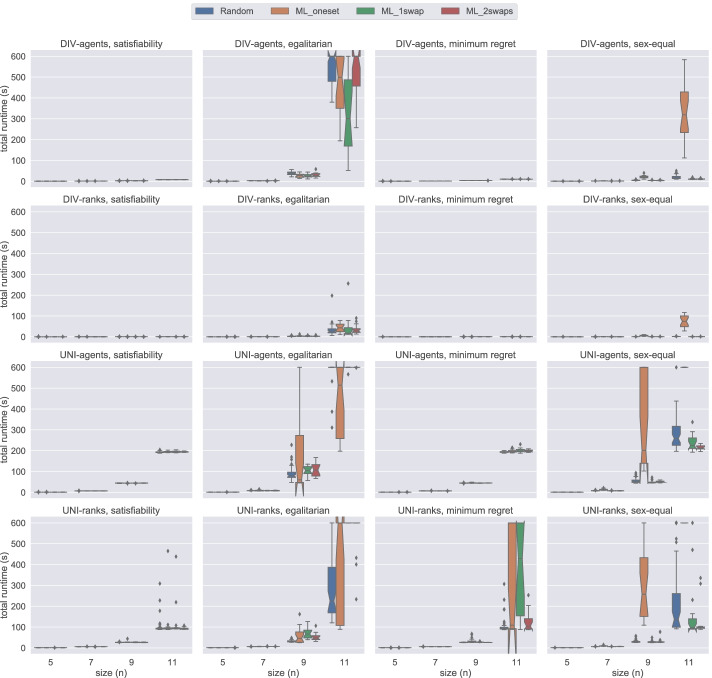
A comparison of total time spent by all models except hs under weak stability using Chuffed

Figure 6 presents a comparison of div-agents, div-ranks, uni-agents, and uni-ranks models on instances of size 5 ≤ *n* ≤ 11 under weak stability. In these plots, we can clearly observe that the div-ranks model has an advantage over the others in terms of total time required to complete the experiments. Contrasting with the findings of Gecode experiments in Fig. 4, where div-agents seems to have an analogous performance with div-ranks, if not better, we observe in Fig. 6 that div-ranks has a clear advantage over div-agents when using Chuffed. We believe this shows that div-ranks benefits greatly from nogood learning. Additionally, using Fig. 6, we can verify our previous observation about ML_oneset being a more challenging dataset generation method for the sex-equal variant under weak stability.

**Fig. 7:**
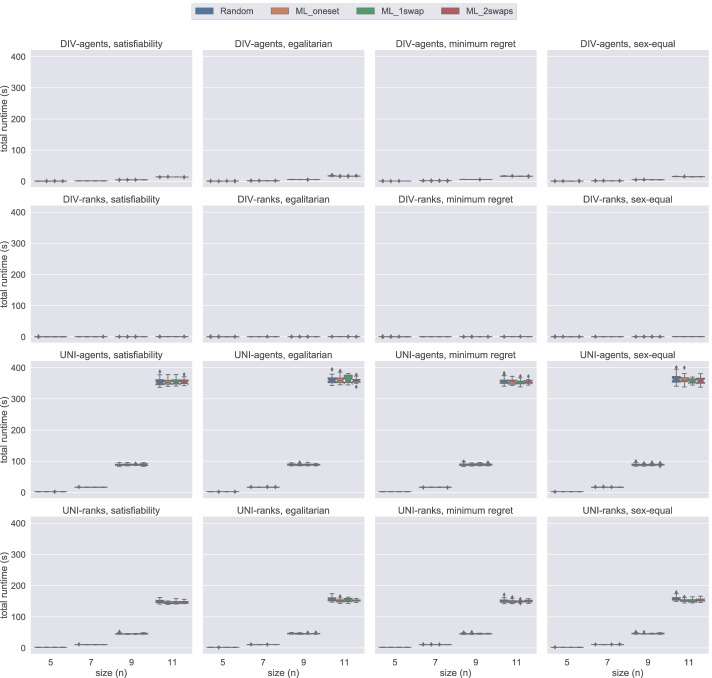
A comparison of total time spent by all models except hs under strong stability using Chuffed

Lastly, Fig. 7 demonstrates a comparison of div-agents, div-ranks, uni-agents, and uni-ranks models on instances of size 5 ≤ *n* ≤ 11 under strong stability. A very straightforward intuition of these tests is that the uni-agents and uni-ranks models are not able to scale well to larger instances. On the other hand, we observe that div-agents handles an increase in the number of agents better than the uni models, but it still performs worse than div-ranks when *n* ≥ 9. Considering the stable and rapid performance of div-ranks using Chuffed and also combining this with our observation in Fig. 5, we conclude that the div-ranks model is the best one to solve 3dsm-cyc under strong stability.

### Scalability

Considering that div-ranks with Chuffed seems to be the best performing combination, we conducted further experiments using this model and solver on instances with *n* ∈{20,23,26,29,32,35,40,45,50,60,70,80,90,100,110,120,130}.

**Fig. 8:**
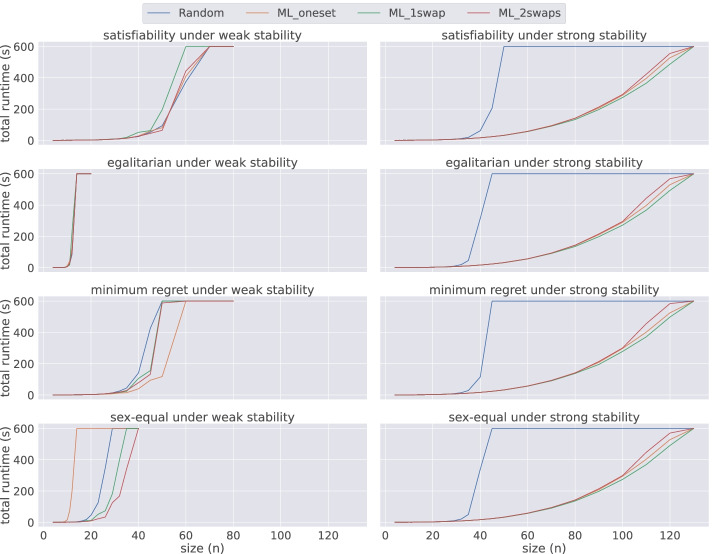
An overview of the performance of div-ranks using Chuffed under both weak and strong stability when solving problem instances of different sizes for each dataset

Figure 8 presents a comparison of the median total time required by div-ranks using freefailsplit strategy on all four datasets both under weak and strong stability. An interesting insight from this figure is that all four problem variants (i.e. satisfiability, egalitarian, minimum regret, and sex-equal) result in identical performances under strong stability, where instances in Random require the longest time to be solved and ML_1swap requires the least. However, we cannot make such a generalisation for weakly stable matchings. For instance, ML_oneset dataset is the most challenging dataset for the sex-equal problem variant, but it is also the least challenging for the minimum regret variant under weak stability.

We present in Table 3 a comparison of the largest sizes reached depending on whether an activity-based heuristic was used. We include three heuristics in the table. First failmin, which is the best performing heuristic not based on activity. For a direct comparison we then have freefailmin, which is the same as failmin except that the solver, Chuffed, is allowed to ignore any search annotations. Finally freefailsplit, which is the one that we chose for Fig. 8 because it performs slightly better than freefailmin.

**Table 3:** Scalability of activity-based heuristics against the best non activity-based heuristic using the DIV-ranks model and the Chuffed solver

Dataset	Problem	Largest size solved before timeout		
		failmin	freefailmin	freefailsplit
Random	weak, satisfiability	60	60	60
	weak, egalitarian	12	12	12
	weak, minimum regret	40	**45**	**45**
	weak, sex-equal	20	**26**	**26**
	strong, satisfiability	45	45	45
	strong, egalitarian	40	40	40
	strong, minimum regret	40	40	40
	strong, sex-equal	40	40	40
ML_oneset	weak, satisfiability	60	60	60
	weak, egalitarian	12	12	12
	weak, minimum regret	**70**	60	50
	weak, sex-equal	12	12	12
	strong, satisfiability	120	120	120
	strong, egalitarian	120	120	120
	strong, minimum regret	120	120	120
	strong, sex-equal	120	120	120
ML_1swap	weak, satisfiability	23	**50**	**50**
	weak, egalitarian	12	12	12
	weak, minimum regret	23	**50**	45
	weak, sex-equal	20	**32**	**32**
	strong, satisfiability	110	**120**	**120**
	strong, egalitarian	110	**120**	**120**
	strong, minimum regret	110	**120**	**120**
	strong, sex-equal	110	**120**	**120**
ML_2swaps	weak, satisfiability	32	50	**60**
	weak, egalitarian	12	12	12
	weak, minimum regret	26	**50**	**50**
	weak, sex-equal	29	**35**	**35**
	strong, satisfiability	110	**120**	**120**
	strong, egalitarian	100	**120**	**120**
	strong, minimum regret	100	110	**120**
	strong, sex-equal	100	**120**	**120**

Best performance for each problem configuration in bold

For Random and ML_oneset instances, whether an activity-based heuristic is used does not affect performance much. On the other hand, we observe considerable improvements with these heuristics for ML_1swap and ML_2swaps instances. The most remarkable cases are the satisfiability and minimum regret versions of weak stability, where we can solve instances with twice as many variables with the free search option. For seven out of eight (satisfiability, optimisation) combinations, activity-based heuristics increase the maximum size of solved ML_1swap and ML_2swaps instances. Even for the remaining combination, (weak, egalitarian), it is possible that an improvement exists, but the increase in difficulty is too sharp for it to be visible with a ten-minute timeout.

### The sweak metaheuristic

As said before, strongly stable matchings are also weakly stable. We observed in Section 4.4 that strongly stable matchings are found faster when compared to their weak versions. This observation leads to an intuitive question: “When the objective is to find a stable matching under weak stability, is there any benefit in searching for a matching under strong stability first, and, if not found, searching under weak stability?”. We refer to this method as *sweak*, i.e. (strong-)weak stability.

In order to implement sweak we added a Boolean decision variable that is 1 or 0 depending on whether we are enforcing strong stability or not. Before branching on the other decision variables, we branch on this variable first and try setting the variable to 1 first. Notice that, in theory, this approach works for both satisfaction and optimisation as in the latter case the cost obtained under strong stability can serve as an upper bound of the cost under weak stability. In practice, we observe that sweak is mostly suitable for satisfaction, as we show next.

**Fig. 9:**
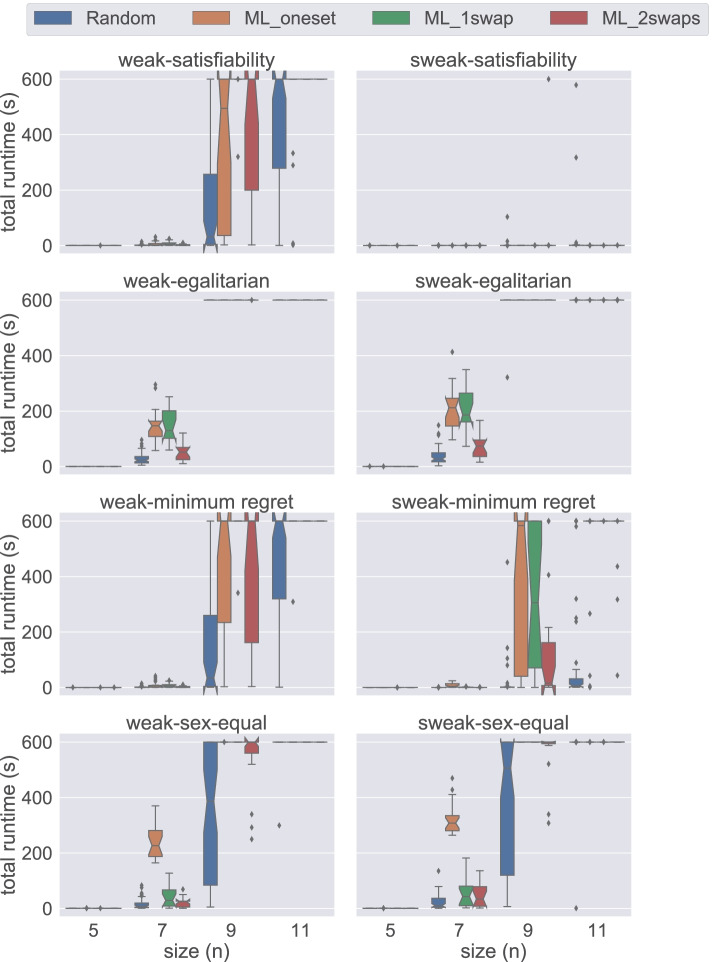
A comparison of the weak and sweak approaches for the DIV-ranks model and the Gecode solver

**Fig. 10:**
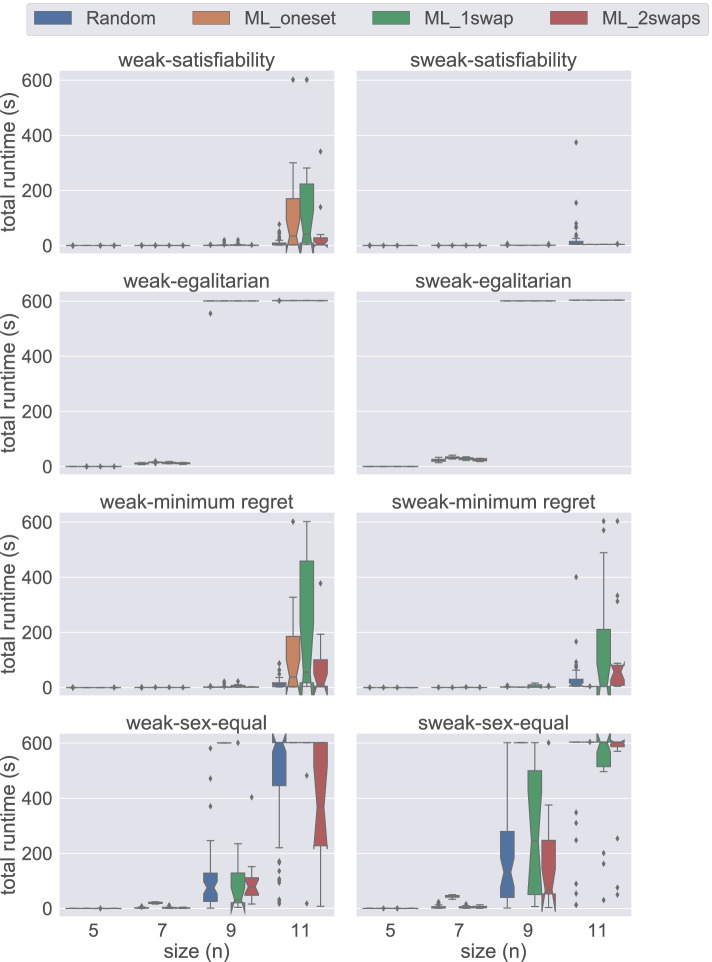
A comparison of the weak and sweak approaches for the HS model and the Gecode solver

**Fig. 11:**
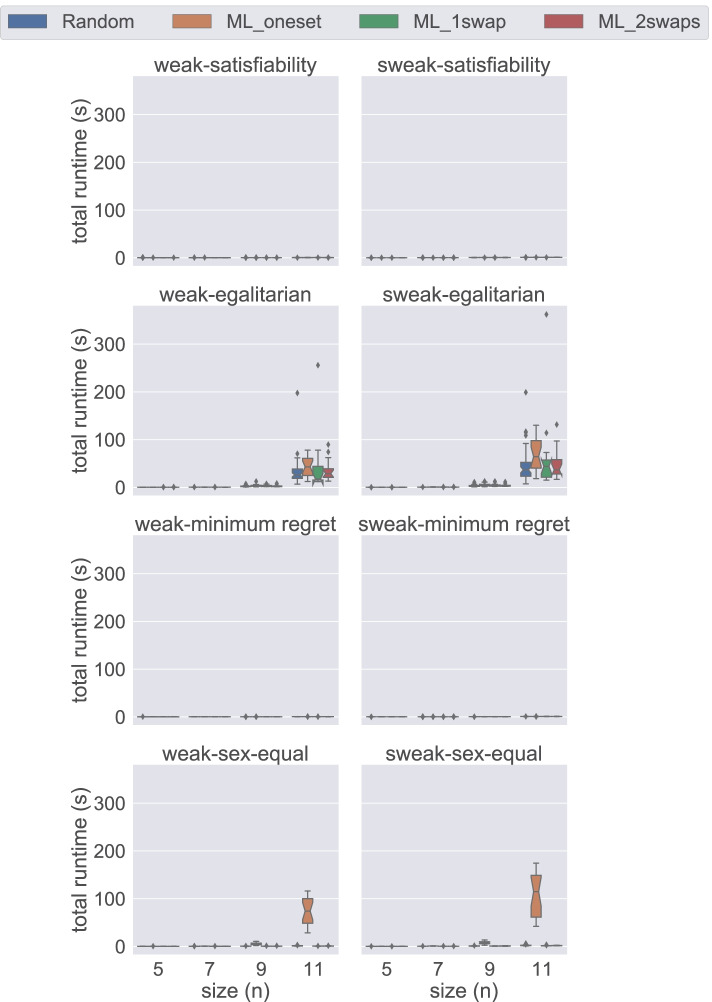
A comparison of the weak and sweak approaches for the DIV-ranks model and the Chuffed solver

**Fig. 12:**
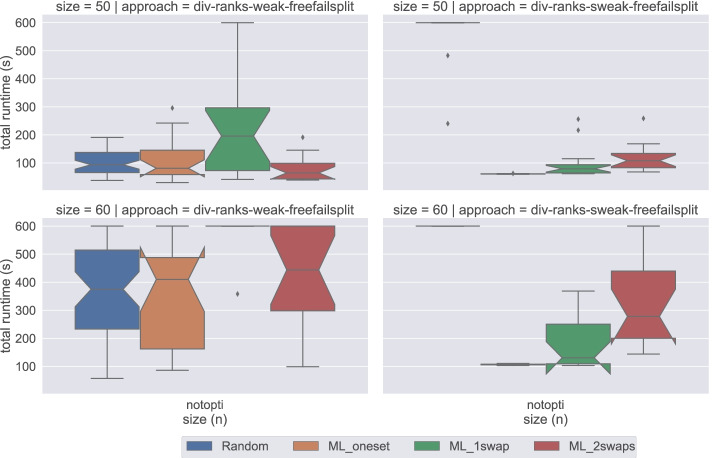
A comparison of the weak and sweak approaches on big satisfiability instances for the DIV-ranks model and the Chuffed solver

**Fig. 13:**
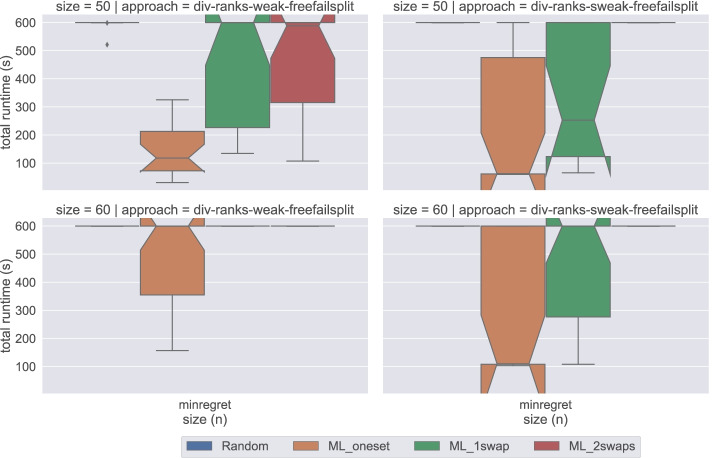
A comparison of the weak and sweak approaches on big minimum regret instances for the DIV-ranks model and the Chuffed solver

In Figs. 9, 10, 11, 12, and 13, we compare the original method to compute weak stability (weak) against the described alternative approach (sweak). Our first observation is that there is significant benefit when solving satisfiability instances when using Gecode. We do not witness such benefit with Chuffed when dealing with small instances. However, we observe some improvement when considering big non-random instances (see Fig. 12). There seems to be no real benefit in the optimisation cases—except in the minimum regret cases, where we observe an improvement even with Chuffed (see Fig. 13).

### Model comparison by number of failures

**Fig. 14:**
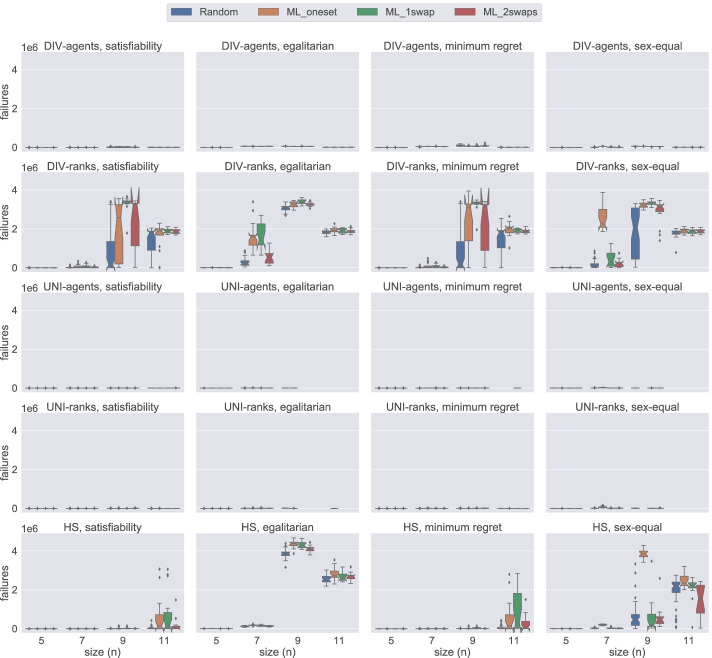
A comparison of the number of failures under weak stability on small instances using Gecode

**Fig. 15:**
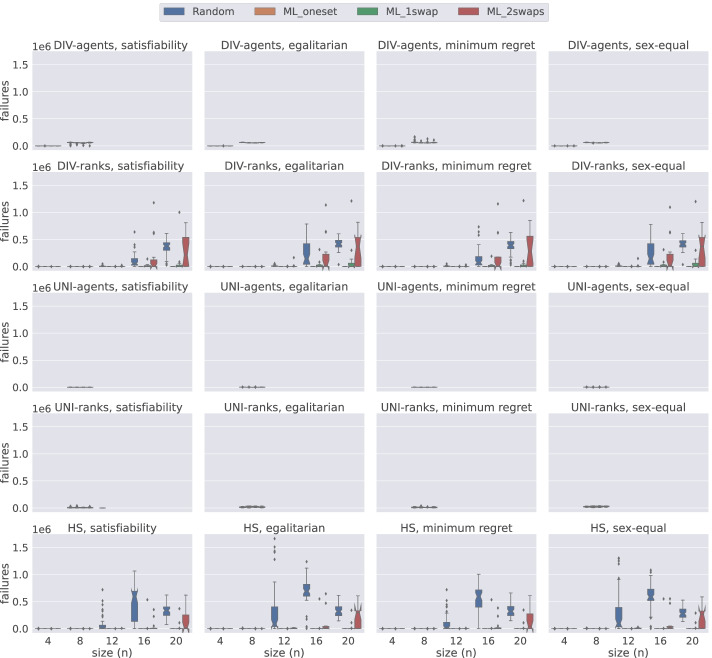
A comparison of the number of failures under strong stability on small instances using Gecode

**Fig. 16:**
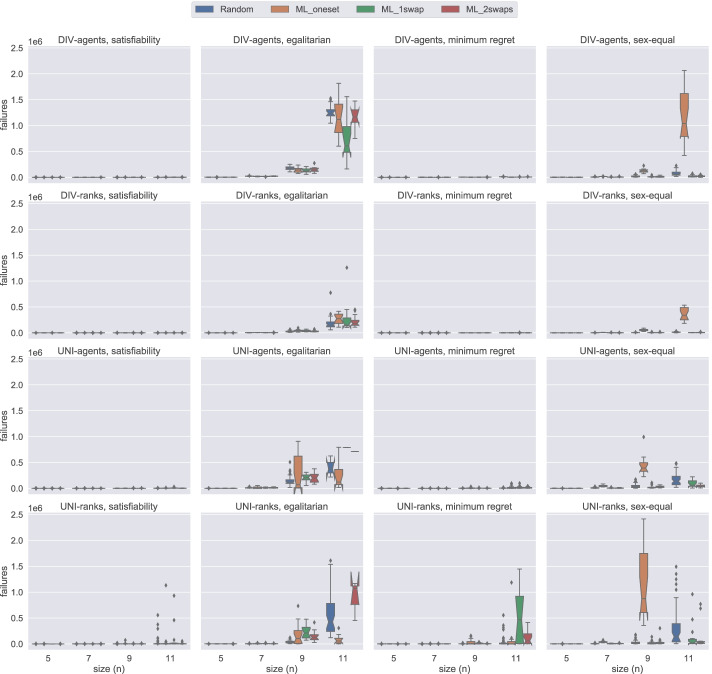
A comparison of the number of failures except hs under weak stability on small instances using Chuffed

**Fig. 17:**
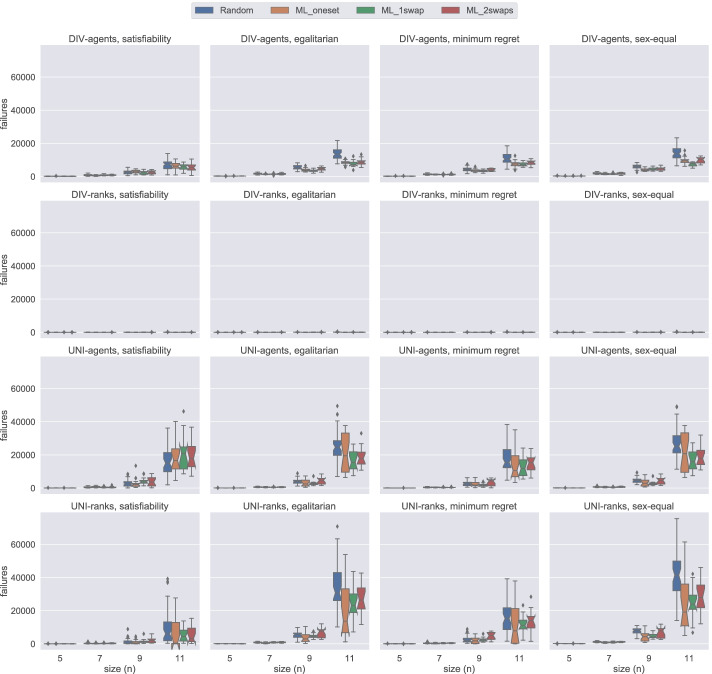
A comparison of the number of failures except hs under strong stability on small instances using Chuffed

In this section, using Figs. 14, 15, 16, and 17, we present the number of failures that the different models have when using the two solvers under the two notions of stability considered. In general, there is a correlation between the number of failures and the total time that a model uses. However, it is important to remark that the number of failures per unit of time (i.e. speed of exploration) is not the same for all models.

One remarkable example has to do with the uni model when using Gecode. The number of failures obtained with this model is very low in most of the cases, see Figs. 14 and 15. However, we end up running out of time with this model since propagation is very expensive, which leads to a very low speed of exploration. This situation is not only observed in uni but also in div-agents.

As expected, for those cases where the time limit has been reached, there is an inverse correlation between the number of failures and the size of the instances. The bigger the instance the more expensive propagation is. This is why, for instance, we observe that for weak stability, hs reports more failures in the egalitarian cases for *n* = 9 than for *n* = 11 (see Fig. 14).

We found a dependency between the number of failures and the solver used. For instance, if we look at the number of failures of div-ranks and uni-ranks when using Gecode on the weak stability instances, div-ranks tends to fail more than uni-ranks. However, in Chuffed, we observe the opposite situation. Our guess is that, somehow, Chuffed is able to take more advantage of the way how constraints are modelled in div-ranks for the generation of nogoods. However, we believe this topic may benefit from further investigation.

Additionally, in Fig. 17, we can clearly observe that div-ranks model using Chuffed under strong stability results in less failures when compared to other configurations, and is quite scalable on the small instances. This observation supports our decision in Sections 4.3 and 4.4 for selecting div-ranks as the best performing configuration.

**Fig. 18:**
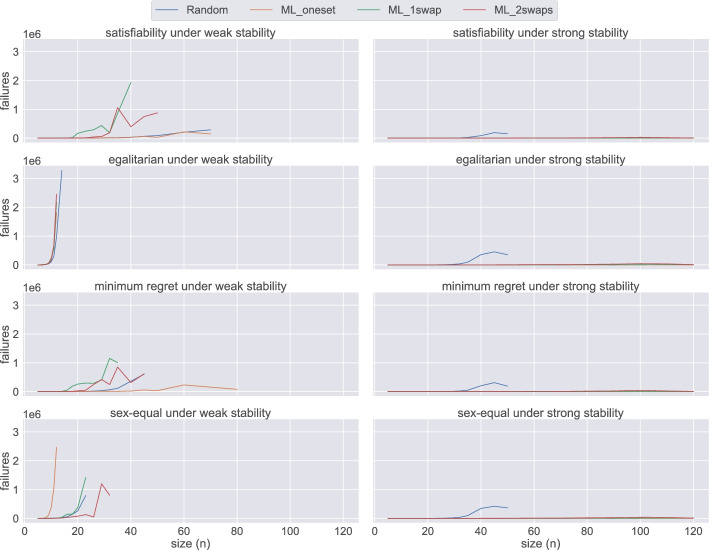
An overview of the number of failures of div-ranks using Chuffed under both weak and strong stability when solving problem instances of different sizes for each dataset

In the plot reporting the scalability (with respect to the number of failures) of div-ranks in the different scenarios (Fig. 18), we observe more clearly how easy ML instances are under strong stability when compared to its weak counterpart. From Lemma 1, we know that the satisfiability problem can be solved in polynomial time for ML_oneset. This also means that the minimum-regret problem variant can be solved in polynomial time. We can observe in Fig. 18 that we are able to solve these instances almost without failing. The complexity of the other two optimisation cases still remains open. However, we can see that the behaviour is pretty much the same. In fact, the same observation holds for ML_1swap and ML_2swaps.

## Conclusion and future work

We proposed a collection of Constraint Programming models to solve the 3-dimensional stable matching problem with cyclic preferences (3dsm-cyc) using both strong and weak stability notions. Additionally, we extended some well-known fairness notions (egalitarian, minimum regret, and sex-equal) to 3dsm-cyc. The five proposed models are fundamentally different from each other in terms of their commitment (individual or group), and also their domain values (agents or ranks). Our experiments show that nogood learning benefits some models more than others, and that the performance of the best models can be further improved by activity-based search heuristics. An unexpected observation is that strong stability turns out to be easier to solve than weak stability. Based on this result, we proposed a new approach to solve the weak stability version of the problem, and showed that it leads to reduced runtimes, at least for satisfiability instances.

Future work could extend our models to more types of instances, for example by allowing preference lists to be incomplete. One could also look at other redundant constraints in order to take most advantage of the properties that some instances exhibit with regard to fairness objectives. We remarked that hs pays a high price for the generation of the BT sets but it is possible to generate the sets by demand instead of doing it eagerly.

## References

[1] Abraham DJ, Levavi A, Manlove DF, O’Malley G (2008). The stable roommates problem with globally-ranked pairs. Internet Mathematics.

[2] Alkan, A. (1988). Nonexistence of stable threesome matchings. *Mathematical Social Sciences,**16*(2), 207–209. https://www.sciencedirect.com/science/article/pii/0165489688900534. Accessed May 2022.

[3] Balinski, M., & Sönmez, T. (1999). A tale of two mechanisms: Student placement. *Journal of Economic Theory*, *84*(1), 73–94. https://www.sciencedirect.com/science/article/pii/S0022053198924693. Accessed May 2022.

[4] Biró, P., Irving, R.W., & Schlotter, I. (2011). Stable matching with couples: An empirical study. *Journal of Experimental Algorithmics (JEA), 16*. 10.1145/1963190.1970372.

[5] Biró P, McDermid E (2010). Three-sided stable matchings with cyclic preferences. Algorithmica.

[6] Boros E, Gurvich V, Jaslar S, Krasner D (2004). Stable matchings in three-sided systems with cyclic preferences. Discrete Mathematics.

[7] Braun, S., Dwenger, N., & Kübler, D. (2010). Telling the truth may not pay off: An empirical study of centralized university admissions in Germany. *The B.E. Journal of Economic Analysis and Policy, 10*(1). 10.2202/1935-1682.2294.

[8] Chen Y, Sönmez T (2002). Improving efficiency of on-campus housing: An experimental study. American Economic Review.

[9] Chu, G. (2011). Improving combinatorial optimization. University of Melbourne.

[10] Clercq SD, Schockaert S, Cock MD, Nowé A (2016). Solving stable matching problems using answer set programming. Theory and Practice of Logic Programming.

[11] Cseh, Á., Escamocher, G., Genç, B., & Quesada, L. (2021). A collection of constraint programming models for the three-dimensional stable matching problem with cyclic preferences. In L.D. Michel (Ed.) *27th International conference on principles and practice of Constraint Programming, CP 2021, Montpellier, France (Virtual Conference), October 25-29, 2021*. Schloss Dagstuhl - Leibniz-Zentrum für Informatik. 10.4230/LIPIcs.CP.2021.22, (Vol. 210 pp. 22:1–22:19).

[12] Cui L, Jia W (2013). Cyclic stable matching for three-sided networking services. Computer Networks.

[13] Danilov VI (2003). Existence of stable matchings in some three-sided systems. Mathematical Social Sciences.

[14] Drummond, J., Perrault, A., & Bacchus, F. (2015). SAT is an effective and complete method for solving stable matching problems with couples. In *Proceedings of the twenty-fourth international joint conference on artificial intelligence, IJCAI 2015, Buenos Aires, Argentina, July 25-31, 2015* (pp. 518–525). AAAI Press.

[15] Eirinakis P, Magos D, Mourtos I, Miliotis P (2012). Finding all stable pairs and solutions to the many-to-many stable matching problem. INFORMS Journal on Computing.

[16] Eriksson K, Sjöstrand J, Strimling P (2006). Three-dimensional stable matching with cyclic preferences. Mathematical Social Sciences.

[17] Escamocher, G., & O’Sullivan, B. (2018). Three-dimensional matching instances are rich in stable matchings. In *Integration of constraint programming, artificial intelligence, and operations research - 15th international conference, CPAIOR 2018, Delft, The Netherlands, June 26-29, 2018, Proceedings*, (Vol. 10848 pp. 182–197). Springer.

[18] Feder T (1992). A new fixed point approach for stable networks and stable marriages. Journal of Computer and System Sciences.

[19] Feder T (1994). Network flow and 2-satisfiability. Algorithmica.

[20] Gai, A., Lebedev, D., Mathieu, F., de Montgolfier, F., Reynier, J., & Viennot, L. (2007). Acyclic preference systems in P2P networks. In *Euro-Par 2007, parallel processing, 13th international euro-par conference, Rennes, France, August 28-31, 2007, Proceedings*, (Vol. 4641 pp. 825–834). Springer.

[21] Gale D, Shapley LS (2013). College admissions and the stability of marriage. The American Mathematical Monthly.

[22] Gecode Team. (2019). Gecode: Generic constraint development environment. Available from: http://www.gecode.org. Accessed May 2022.

[23] Gent, I.P., Irving, R.W., Manlove, D.F., Prosser, P., & Smith, B.M. (2001). A constraint programming approach to the stable marriage problem. In *Principles and practice of constraint programming - CP 2001, 7th international conference, CP 2001, Paphos, Cyprus, November 26 - December 1, 2001, Proceedings*, (Vol. 2239 pp. 225–239). Springer.

[24] Gusfield D (1987). Three fast algorithms for four problems in stable marriage. SIAM Journal on Computing.

[25] Gusfield, D., & Irving, R.W. (1989). *The stable marriage problem - structure and algorithms*. MIT Press.

[26] Halldórsson MM, Irving RW, Iwama K, Manlove DF, Miyazaki S, Morita Y, Scott S (2003). Approximability results for stable marriage problems with ties. Theoretical Computer Science.

[27] Huang, C. (2007). Two’s company, three’s a crowd: Stable family and threesome roommates problems. In *Algorithms - ESA 2007, 15th annual european symposium, Eilat, Israel, October 8-10, 2007, proceedings*, (Vol. 4698 pp. 558–569). Springer.

[28] Irving RW (1985). An efficient algorithm for the “stable roommates” problem. Journal of Algorithms.

[29] Irving RW, Leather P, Gusfield D (1987). An efficient algorithm for the “optimal” stable marriage. Journal of Algorithms.

[30] Iwama, K., & Miyazaki, S. (2008). A survey of the stable marriage problem and its variants. In *International conference on informatics education and research for knowledge-circulating society (ICKS 2008)* (pp. 131–136).

[31] Kato A (1993). Complexity of the sex-equal stable marriage problem. Japan Journal of Industrial and Applied Mathematics.

[32] Knuth, D.E. (1976). Mariages stables. Les Presses de L’Université de Montréal. (English translation in *Stable Marriage and its Relation to Other Combinatorial Problems*, volume 10 of CRM Proceedings and Lecture Notes, American Mathematical Society, 1997).

[33] Lam, C., & Plaxton, C.G. (2019). On the existence of three-dimensional stable matchings with cyclic preferences. In *Algorithmic game theory - 12th international symposium, SAGT 2019, Athens, Greece, September 30 - October 3, 2019, proceedings*, (Vol. 11801 pp. 329–342). Springer.

[34] Manlove, D.F. (2013). *Algorithmics of matching under preferences*, vol. 2. WorldScientific.

[35] Manlove, D.F., O’Malley, G., Prosser, P., & Unsworth, C. (2007). A constraint programming approach to the hospitals / residents problem. In *Integration of AI and OR techniques in constraint programming for combinatorial optimization problems, 4th international conference, CPAIOR 2007, Brussels, Belgium, May 23-26, 2007, Proceedings*, (Vol. 4510 pp. 155–170). Springer.

[36] McDermid E, Irving RW (2014). Sex-equal stable matchings: Complexity and exact algorithms. Algorithmica.

[37] Mcgill R, Tukey JW, Larsen WA (1978). Variations of box plots. The American Statistician.

[38] Nethercote, N., Stuckey, P.J., Becket, R., Brand, S., Duck, G.J., & Tack, G. (2007). Minizinc: Towards a standard CP modelling language. In *Principles and practice of constraint programming - CP 2007, 13th international conference, CP 2007, Providence, RI, USA, September 23-27, 2007, proceedings*, (Vol. 4741 pp. 529–543). Springer.

[39] Ng C, Hirschberg DS (1991). Three-dimensional stable matching problems. SIAM Journal on Discrete Mathematics.

[40] O’Malley, G. (2007). *Algorithmic aspects of stable matching problems* (Unpublished doctoral dissertation). University of Glasgow, UK.

[41] Panchal, N., & Sharma, S. (2014). An efficient algorithm for three dimensional cyclic stable matching. *International Journal of Engineering Research and Technology, 3*(4).

[42] Pashkovich K, Poirrier L (2020). Three-dimensional stable matching with cyclic preferences. Optimization Letters.

[43] Perach N, Polak J, Rothblum UG (2008). A stable matching model with an entrance criterion applied to the assignment of students to dormitories at the Technion. International Journal of Game Theory.

[44] Raveendran, N., Zha, Y., Zhang, Y., Liu, X., & Han, Z. (2019). Virtual core network resource allocation in 5G systems using three-sided matching. ICC 2019-2019 IEEE international conference on communications (ICC), pp. 1–6.

[45] Roth AE, Sotomayor MAO (1990). Two-sided matching: A study in game-theoretic modeling and analysis.

[46] Siala, M., & O’Sullivan, B. (2016). Revisiting two-sided stability constraints. In *International conference on ai and or techniques in constraint programming for combinatorial optimization problems* (pp. 342–357).

[47] Siala, M., & O’Sullivan, B. (2017). Rotation-based formulation for stable matching. In *International conference on principles and practice of constraint programming* (pp. 262–277).

[48] Stuckey, P.J., Marriott, K., & Tack, G. (2021). The minizinc handbook v2.5.5. Last Accessed on 22 Dec 2021.

[49] Subramanian A (1994). A new approach to stable matching problems. SIAM Journal on Computing.

[50] Unsworth, C., & Prosser, P. (2005). A specialised binary constraint for the stable marriage problem. In *Abstraction, reformulation and approximation, 6th international symposium, SARA 2005, Airth Castle, Scotland, UK, July 26-29, 2005, Proceedings*, (Vol. 3607 pp. 218–233). Springer.

[51] Unsworth, C., & Prosser, P. (2013). An n-ary constraint for the stable marriage problem. arXiv:1308.0183.

[52] Woeginger, G.J. (2013). Core stability in hedonic coalition formation. In *Proceedings of SOFSEM 2013, 39th international conference on current trends in theory and practice of computer science*, (Vol. 7741 pp. 33–50). Springer.

